# Unsupervised learning-based optimization of college students’ fundamental capacity evaluation method

**DOI:** 10.3389/fphys.2026.1921534

**Published:** 2026-09-22

**Authors:** Lei Jiang, Jinxi Zhang, Youwei Zhang, Yanan Gao, Rufeng Su, Zhixiong Zhou

**Affiliations:** 1 Institute of Physical Education and Training, Capital University of Physical Education and Sports, Beijing, China; 2 Institute of Artificial Intelligence in Sports, Capital University of Physical Education and Sports, Beijing, China; 3 School of Physical Education and Sport Science, Fujian Normal University, Fuzhou, China; 4 Department of Exchange and Cooperation, Capital University of Physical Education and Sports, Beijing, China; 5 Beijing Key Laboratory of Interdisciplinary Intelligent Technology of Sports, Medicine and Engineering, Beijing, China

**Keywords:** college students, entropy weight method, evaluation rule, fundamental capacity screen, unsupervised learning

## Abstract

**Background:**

The Fundamental Capacity Screen (FCS) is a standardized method for assessing athletic performance and fundamental capacity (FC). However, its current framework lacks refinement, yielding qualitative and insufficiently detailed results that hinder precise athletic training and performance evaluation.

**Objective:**

This study employs unsupervised learning to refine FC evaluation, enhancing its objectivity and scientific rigor by providing a more data-driven and interpretable approach.

**Methods:**

Data from 123 college students (61 males, mean age 20.42 ± 1.88 years; 62 females, mean age 20.35 ± 1.85 years) regarding FC test indicators were initially subjected to a factor analysis (FA) model to identify underlying factors. Hierarchical clustering was then performed on the first factor. Based on these results, relevant rules of abilities were established, and principal component analysis (PCA) was employed to reduce the dimensionality of multidimensional indicators. Finally, the entropy weight method was used to calculate and adjust the scores of each ability, and the scores were summarized and classified by percentile.

**Results:**

FA revealed 3 distinct factors (jumping ability, upper limb ability, and lower limb ability) that together explained 0.8569 of the total variance. The hierarchical clustering dendrogram indicated that jumping ability could be further subdivided. Based on this, 5 ability categories were defined: upper limb balance control, lower limb balance control, core-mediated transfer control, explosive control, and impact control. The composite scores were categorized into 5 intervals: poor, pass, good, excellent, and outstanding. The depicted radar map shows the median characteristics of the composite scores of different abilities.

**Conclusions:**

Results demonstrate that unsupervised learning provides a more intuitive and objective framework for FC assessment, offering a novel perspective for athletic performance evaluation and training optimization.

## 1 Introduction

The Fundamental Capacity Screen (FCS) is a theoretical framework derived from the Functional Movement Screen (FMS) by Gray Cook, a famous physical fitness expert, represents an innovative tool for evaluating an individual’s fundamental capacity (FC) (Functional Movement Systems, [[NoYear]]). FCS is designed to bridge the gap between athletic performance and the development of specific skills, offering a systematic approach to identify issues in movement patterns, physical capabilities, and skill-related functions. The tool is comprised of 4 primary components: movement control, postural control, explosive control, and impact control (Functional Movement Systems, [[NoYear]]). The screening process targets individuals with FMS score exceeding 14 points, assessing their capacities across these 4 domains. Each domain is evaluated using a binary yes/no criterion, providing a straightforward measure of the individual’s physical condition (Li et al., 2023). The FCS results offer a comprehensive understanding of the participant’s fitness level and adaptive potential, particularly for youth and adult populations (Functional Movement Systems, [[NoYear]]; Li et al., 2023).

Although the FCS provides a broad evaluation of an individual’s physical fitness, it is not without limitations. Firstly, the categorization of FC within the FCS is overly simplistic, failing to encompass critical elements such as core-mediated transfer control, which is fundamental to overall FC. This limitation suggests that the tool lacks the precision and detail necessary for accurate capability assessment. Secondly, the FCS primarily relies on determinative index, lacking intuitive quantitative data that could more accurately reflect an individual’s FC. The binary yes/no approach is insufficient to capture the subtleties of an individual’s physical capacity. In contrast, a numerical evaluation system would offer a more direct and precise depiction of individual differences in physical abilities. Moreover, postural control is a complex, multi-factorial construct involving sensory integration (visual, vestibular, proprioceptive), cognitive attention, prior experience, and anticipatory/reactive strategies (Chapman et al., 2008; Bourelle et al., 2014; Prosperini et al., 2016; Lim et al., 2019). Simple linear reach or jump distances cannot validly isolate these components; therefore, including posture control agent metrics that are not clearly defined will affect the validity of the evaluation. Consequently, the present study excludes postural control from the quantitative framework while introducing a core-related dimension grounded in the documented role of the core in energy transfer during arm-swing jumps (Hibbs et al., 2008).

To address these issues, the present study introduces a novel unsupervised learning-based evaluation method (Chen et al., 2023; Bruce et al., 2024; Do et al., 2024; Kupers et al., 2024; Lin et al., 2024; Martin et al., 2024; Rotbei et al., 2024). Unsupervised learning aims to learn statistical patterns from training data when no explicit labels are available (Storrs et al., 2021). By conducting in-depth analysis of quantitative data from diverse physical fitness metrics, this approach aims to enhance the accuracy and objectivity of FC evaluation.

This study focused on college students as the research subjects and expanded the FCS framework. It utilized the 8 indicators included in movement control, explosive control, and impact control from the FCS, and further expanded them to 12 indicators. Systematically, it explored the intrinsic relationships among various physical fitness indicators. Using factor analysis (FA) (Zhao et al., 2024), we extracted latent factors encompassing multiple variables, which were subsequently analyzed in conjunction with hierarchical clustering algorithms to quantify inter-indicator distances and enable deeper insights. This innovative approach not only facilitates a more nuanced, multidimensional assessment of FC in college students but also establishes a methodological foundation that can be adapted for FC evaluation in other demographic groups.

We hypothesized that: (1) unsupervised learning (FA combined with hierarchical clustering) applied to quantitative FC indicators will reveal a more refined multi-dimensional structure than the original four binary domains of FCS; and (2) the sequential FA–hierarchical clustering–PCA–EWM pipeline will produce continuous, objectively weighted composite scores and ability profiles that offer greater granularity and practical interpretability than binary FCS outcomes.

## 2 Methods

### 2.1 Study participants

A total of 123 students from college in Beijing, China were recruited as test subjects in this study, including 61 male students (age: 20.42 ± 1.88 years old, height: 177.5 ± 5.69 cm, weight: 71.74 ± 12.71 kg, BMI: 22.44 ± 3.55 kg/m^2^) and 62 female students (age: 20.35 ± 1.85 years, height: 164.42 ± 5.36 cm, weight: 55.5 ± 7.58 kg, BMI: 20.55 ± 2.52 kg/m^2^). All participants were randomly selected through public recruitment.

Inclusion Criteria: Participants were required to be healthy, without a history of disease, and capable of engaging in physical activity; (2) participants had to have refrained from engaging in high-intensity physical activity for two days prior to the test and not be in a state of physical fatigue.

Exclusion Criteria: (1) Students with underlying organ diseases or conditions that contraindicate participation in physical activity; (2) students with abnormal physical development or disabilities.

The study protocol was reviewed and approved by the Ethics Committee of the Capital University of Physical Education and Sports (No.2025A141). Written informed consent was obtained from all participants prior to their involvement in the study.

### 2.2 Data collection and relevant descriptions

This study was based on the FCS and incorporated insights from interviews with 8 experts in sports training from institutions including the Capital University of Physical Education and Sports and Shanghai University of Sport in China. After careful consideration, 8 tests were selected: lower limb extension, upper limb extension, standing long jump, hands-on-hips long jump, single-leg jump, triple jump, double jump, and the 2-1–2 jump. A total of 12 different measured values were collected, as shown in **Table 1** (Li et al., 2023).

**Table 1:** Description of test indicator data.

Type	Indicator	Test method
Lower limb extension	Left leg extension	The right foot was placed on the measurement device’s platform, with the toes aligned with the front edge of the platform, maintaining a single-leg stance. The right knee was then slowly bent, lowering the body to its maximum extent, while the left foot’s toes touched the edge of the sliding board. The left foot was then moved forward, sliding the board as far as possible in a continuous motion. The position was then smoothly returned to the starting posture.
	Right leg extension	The left and right side are opposite to the left leg extension.
Upper limb extension	Left hand extension	The right hand was placed on the platform, with the body positioned at a 45° angle relative to the rear extension line of the platform. The left hand rested on the front edge of the sliding board. The right elbow was slowly bent, bringing the arm down to its fullest extent, while the left fingertip pushed the sliding board forward in a continuous motion until it reached the maximum distance. The body was then smoothly returned to the starting position.
	Right hand extension	The left and right side are opposite to the left hand extension.
Standing long jump	Standing long jump	In a standing posture, the subject jumped forward using both feet.
Hands-on-hips long jump	Hands-on-hips long jump	In a standing posture, with the hands placed on the hips, the subject jumped forward using both feet.
Single-leg jump	Left single-leg jump	The right foot remained planted on the ground while the left leg performed a single-leg jump. During this, the arms naturally swung. After the left foot landed, the jump was completed with the left leg landing alone.
	Right single-leg jump	The left foot remained planted on the ground while the right leg performed a single-leg jump. During this, the arms naturally swung. After the right foot landed, the jump was completed with the right leg landing alone.
Triple jump	Triple jump	In a standing posture, both feet were used to jump forward consecutively 3 times.
Double jump	Double jump	In a standing posture, both feet were used to jump forward consecutively two times.
2-1–2 jump	Left 212 jump	In a standing posture, both feet were used to jump forward (denoted as 2). Then, the left leg landed (denoted as 1), followed by a left single-leg jump, and both feet landed again (denoted as 2).
	Right 212 jump	In a standing posture, both feet were used to jump forward (denoted as 2). Then, the right leg landed (denoted as 1), followed by a right single-leg jump, and both feet landed again (denoted as 2).

All tests were conducted in the exact order listed in **Table 1**, with 2–3 minutes of rest between repeated trials of the same test and approximately 5 minutes of rest between different tests to minimize residual fatigue while maintaining session feasibility. Each test was repeated 3 times, and the best performance for each test was used as the final data point. Lower limb extension and upper limb extension were measured using the FC assessment tool kit, which included a ranging laser head, slide, and slider. The remaining indicators were assessed using a standardized tape measure.

### 2.3 Unsupervised learning

This study aims to construct a comprehensive evaluation model and new methods for FC based on the measured relevant indicators. Therefore, this task belongs to unsupervised learning. At present, the more popular unsupervised learning algorithms include FA, Principal Component Analysis (PCA), clustering algorithms, etc. The methods for calculating the comprehensive score by calculating weights include the Entropy Weight Method (EWM), TOPSIS, etc. In this study, FA, hierarchical clustering, PCA and EWM were used for analysis in sequence.

#### 2.3.1 FA

To categorize the collected indicators, FA was first performed on the standardized dataset of FC related variables. FA is a widely used dimensionality reduction technique, particularly in fields such as social sciences and medicine. The applicability of FA requires that the original variables be significantly correlated. Therefore, Bartlett’s test of sphericity and the Kaiser-Meyer-Olkin (KMO) test were employed to assess the suitability of the data (Herrmann et al., 2019; Lasalvia et al., 2023). Bartlett’s test evaluates whether the variables are correlated; a p-value less than 0.05 indicates that FA is appropriate. The KMO test measures the overall strength of correlations between variables, with values ranging from 0 to 1, higher values indicate stronger correlations. In this study, Bartlett’s test yielded a p-value of 0.0, suggesting that the correlation matrix is not an identity matrix, and that the variables are significantly related, making the data suitable for factor analysis. The KMO value was 0.8963, confirming the strong correlation among the variables. Based on the results of these two tests, the dataset was deemed appropriate for conducting FA.

#### 2.3.2 Hierarchical clustering

Hierarchical clustering is a technique that constructs a dendrogram to hierarchically partition data based on the differences between samples (Harmeling and Williams, 2011). The proximity of nodes in the dendrogram reflects the degree of similarity between them: the closer the nodes, the higher their similarity; conversely, the greater the distance, the lower the similarity. At the lowest level of the dendrogram, each node represents an individual data point, while at the highest level, all nodes converge into a single root node, representing the clustering outcome of the entire dataset. Hierarchical clustering can be implemented through two primary strategies: bottom-up (agglomerative) and top-down (divisive) approaches (Kim et al., 2018). In this study, the bottom-up agglomerative method was employed.

#### 2.3.3 PCA

PCA is a multivariate statistical technique grounded in the concept of dimensionality reduction (Ingram et al., 2024). It transforms multiple correlated variables into a smaller set of uncorrelated composite indicators, known as principal components, while retaining as much of the original information as possible. Each principal component is a linear combination of the original variables, and these components are mutually orthogonal, ensuring independence among them. This transformation not only simplifies the data but also enhances the interpretability by capturing the most significant underlying patterns among the variables, thereby reducing complexity and improving analytical efficiency (Ringnér, 2008).

#### 2.3.4 EWM

EWM determines the weight of each indicator based on its information entropy, where a lower entropy value corresponds to a higher weight (Jin et al., 2024). This method is particularly objective, effectively minimizing the influence of subjective factors, and is especially suited for situations where the correlation between variables is low. The composite score calculated using EWM does not have a fixed value; rather, it is contingent on the selected indicators and their respective values. EWM primarily serves to assign weights to each indicator, while the composite score is derived from the weighted sum of the indicators’ actual values. Both the weight and the corresponding value of each indicator affect the final composite score (Luo et al., 2022).

The formula for information entropy calculation is presented in **Equation 1**:

(1) $$H\left(X\right)=\;-\sum_{i=1}^{q}p\left(x_{i}\right)\log\left(p\left(x_{i}\right)\right)$$

Where *H* represents the information entropy, *q* denotes the number of messages in the source, and *p*(*x_i_*) is the probability of the occurrence of message *x_i_*.

Information entropy serves as a measure of the disorder or uncertainty within a system. According to its definition, the entropy value of a given indicator can be used to assess its degree of dispersion. A smaller entropy value indicates greater dispersion of the indicator, thus resulting in a larger weight in the overall evaluation. Conversely, if all values of a given indicator are equal, it exerts no influence on the composite evaluation. Therefore, information entropy provides an effective tool for calculating the weight of each indicator, thereby offering a scientific basis for multi-indicator comprehensive evaluation.

The detailed calculation steps are presented in **Equations 2**–**6** (Jin et al., 2024):

(1) Data normalization;

(2) $$X_{ij}=\frac{x_{ij}-\min\left(x_{i}\right)}{\max\left(x_{i}\right)-\min\left(x_{i}\right)}$$

(2) Calculation of proportions;

(3) $$p_{ij}=\frac{x_{ij}}{\sum_{i=1}^{m}x_{ij}}$$

(3) Calculation of entropy values;

(4) $$E_{j}=-k*\sum_{i=1}^{n}\left(p_{ij}*\ln\left(p_{ij}\right)\right)$$

(4) Calculation of weights;

(5) $$\omega_{j}=\frac{1-E_{j}}{\sum_{j=1}^{m}\left(1-E_{j}\right)}$$

(5) Calculation of the composite score.

(6) $$S_{i}=\sum_{j=1}^{m}\left(\omega_{j}*X_{ij}\right)$$

Among others, the above: *i* = 1, 2, …, *n*; *j* = 1, 2, …, *m*.

### 2.4 Classification of composite scores

After the composite-score calculation, five-level classification cut-off values were formulated with reference to the 100-point evaluation framework of the *National Student Physical Health Standard (Revised 2014)* of China. This official standard sets 60 points as the pass threshold and adopts a four-grade classification system. Two rounds of expert-panel consultation were conducted to finalize and refine the boundaries for the five-level grading used in the present study. The resulting classification criteria were Poor (< 60), Pass (60-70), Good (70-85), Excellent (85-95), Outstanding (95-100). It should be noted that these thresholds are study-specific modifications and are not equivalent to the official national normative criteria.

### 2.5 Statistical analysis

Already all data analyses were conducted using Python version 3.7.0 (Fredericksburg, VA, USA). The radar chart was completed through the ECharts visualization website (https://echarts.apache.org).

## 3 Results

### 3.1 FA results

The standardized data for the relevant test indicators were used to construct the correlation matrix heatmap and density distribution histograms for each variable, as shown in **Figure 1**. The upper triangular correlation coefficient heatmap reveals strong inter-variable relationships, with the majority of coefficients exceeding 0.5 and most surpassing 0.8. The test indicators can be broadly categorized into 3 groups. The correlation coefficients between the first 8 variables are generally above 0.8, with some even surpassing 0.9. Specifically, the correlation coefficient between left and right hand extension is 0.86, while that between left and right leg extension is 0.85. Moreover, the distribution plots of the variables along the diagonal suggest that the majority of the variables exhibit a near-normal distribution, with a relatively uniform spread.

**Figure 1:**
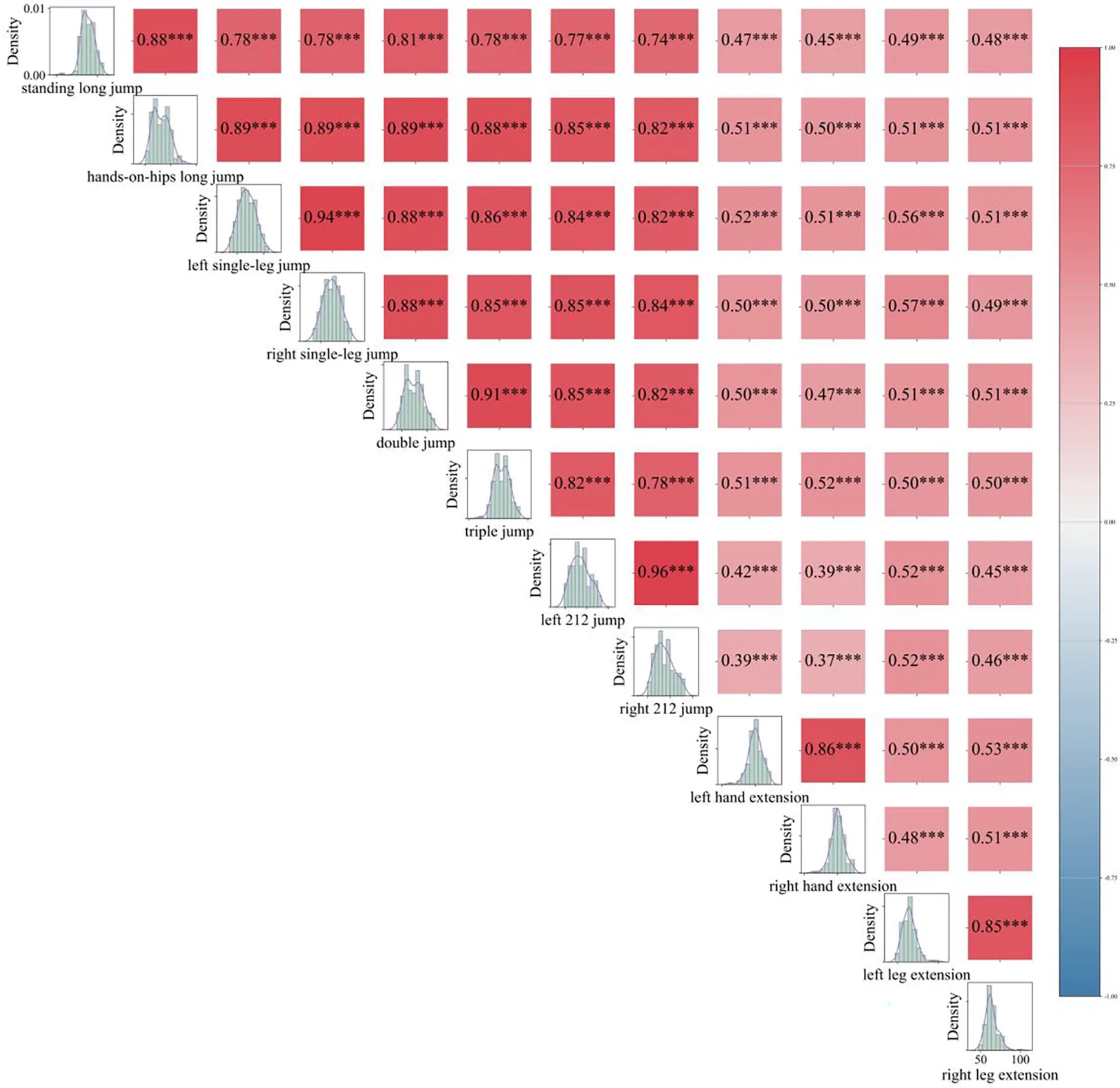
Heat map of correlation coefficients of FC test indicators.

#### 3.1.1 Factor score and expression

As illustrated in **Figure 2A**, the scree plot reveals a noticeable decline when the number of factors reaches 3. Based on this observation, 3 factors were selected for the analysis.

**Figure 2:**
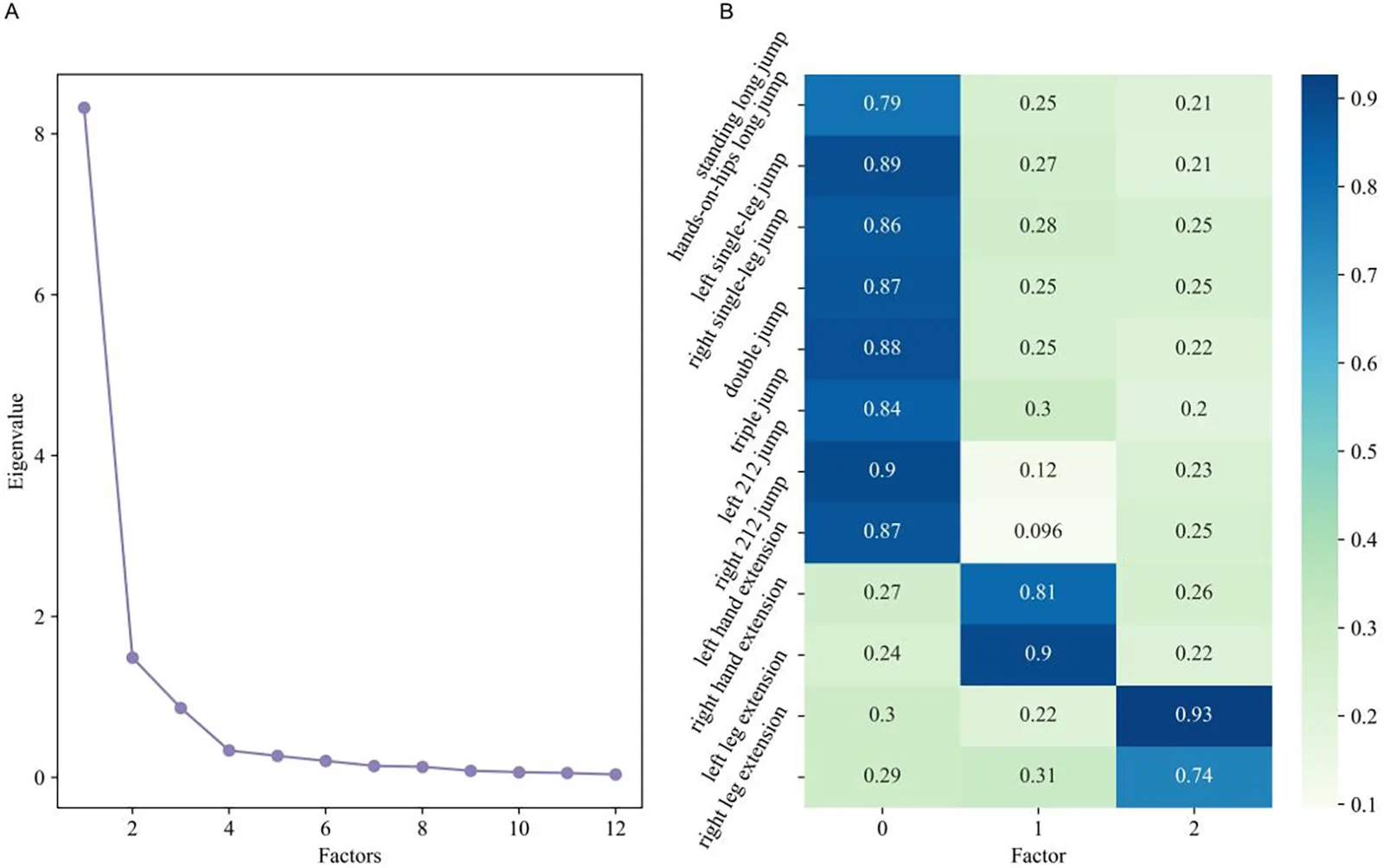
Partial results of FA. **(A)** scree plot, **(B)** heat map of rotated component matrix.

The rotated component matrix is depicted in the heatmap shown in **Figure 2B**. In FA, if the extracted factors have clear interpretations, the model’s explanatory power and practical relevance are significantly enhanced. The factor loading matrix was rotated to ensure that each variable had a large loading on only one common factor, while the loadings on the other factors remained minimal. The maximum variance rotation method within orthogonal rotation was applied in this study. The results indicate that, under a three-factor model, the first factor contributes the most (0.5221), followed by the second factor (0.1719) and the third factor (0.1629). The cumulative variance explained by the 3 factors is 0.8569, demonstrating the robustness of the model.

In the factor analysis, the following tests were assigned to the first factor, which corresponds to jumping ability: standing long jump, hands-on-hips long jump, left single-leg jump, right single-leg jump, double jump, triple jump, left 212 jump, and right 212 jump. The second factor, associated with upper limb ability, was composed of the left and right arm reach. The third factor, reflecting lower limb ability, was represented by the left and right leg reach.

Based on this factor structure, we calculated composite scores for each factor. The mathematical expressions for the 3 common factors (*F*_1_, *F*_2_, *F*_3_) were derived from the factor-score coefficient matrix obtained by the regression method after varimax rotation (distinct from the factor loadings shown in **Figure 2B**, which represent correlations between observed variables and latent factors). Results from the formulas are presented in **Equations 7**–**9**.

(7) $$F_{1}=0.0359X_{1}+0.2338X_{2}+0.1255+\;0.2278X_{4}+0.1795X_{5}+0.0864X_{6}+\;0.3267X_{7}+0.0435X_{8}-0.0731X_{9}-0.1562X_{10}-0.2568X_{11}+0.0096X_{12}$$

(8) $$F_{2}=0.0034X_{1}-\;0.0008X_{2}+0.0335X_{3}-\;0.0452X_{4}+0.0311X_{5}-0.0219X_{6}-\;0.1205X_{7}-0.1006X_{8}+0.3044+0.8138X_{10}-0.2483X_{11}-0.0465X_{12}$$

(9) $$F_{3}=-0.0292X_{1}+\;0.0209X_{2}-0.0742X_{3}-\;0.2176X_{4}+0.0355X_{5}-0.0199X_{6}-\;0.2041X_{7}+0.1202X_{8}-0.0376X_{9}-0.1437X_{10}+1.2720X_{11}-0.0644X_{12}$$

These factor scores provide a quantitative representation of the underlying physical abilities, allowing for more precise analysis of individual FC profiles.

#### 3.1.2 FA composite score model

By substituting the indicator data into the factor score equations, the overall composite scores were calculated, followed by a comprehensive analysis and evaluation. The final FA composite score model was constructed by performing a linear weighted average, using the factor scores and the variance explained by each factor as the respective weights, as shown in **Equation 10**:

(10) $$F=\frac{0.5221}{0.8569}F_{1}+\;\frac{0.1719}{0.8569}F_{2}+\frac{0.1629}{0.8569}F_{3}$$

The FA composite score model provides a robust and scientifically validated framework for comprehensive fitness assessment. By simultaneously considering the 3 critical dimensions of physical performance—jumping ability, upper limb ability, and lower limb ability. The weighted integration of these factors ensures that the relative importance of each physical attribute is appropriately represented in the final assessment, thereby enhancing the model’s practical utility for both research and applied settings.

### 3.2 Comprehensive analysis results

#### 3.2.1 The results of Hierarchical clustering and PCA

In the FA results, the hierarchical clustering dendrogram of the first factor’s test indicators is presented in **Figure 3**. Based on this FA model, we can make an initial evaluation of the FC using the basic indicators related to physical movement functions. Furthermore, given the complexity and multiplicity of test indicators associated with jumping ability, we conducted a dedicated hierarchical cluster analysis to further refine this category. By calculating pairwise distances between each test indicator, we generated a corresponding hierarchical clustering dendrogram, which provides a systematic visualization of the relationships and groupings among these variables. This approach not only enhances the interpretability of the results but also offers a more granular understanding of the underlying structure of jumping ability metrics.

**Figure 3:**
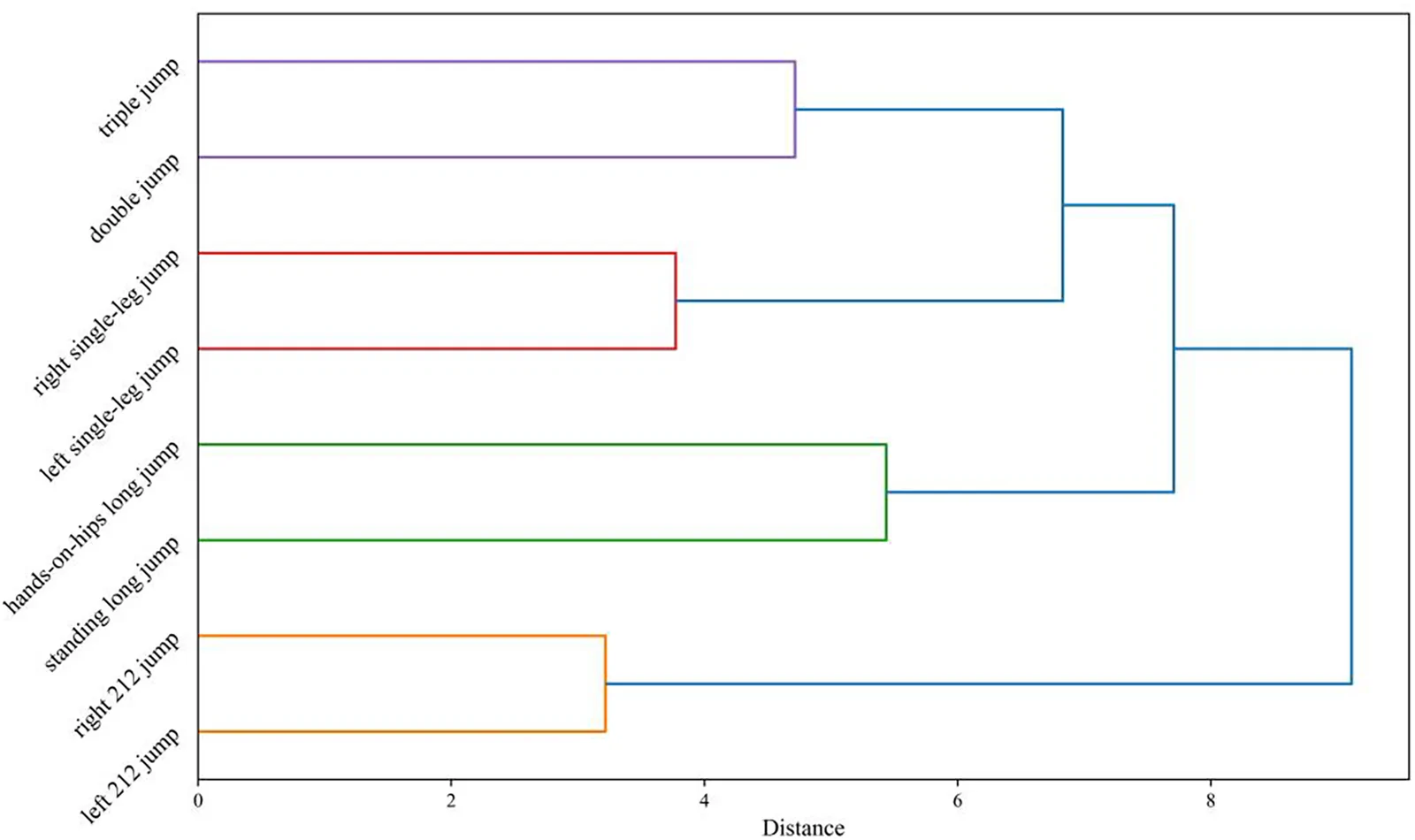
Hierarchical clustering tree of first factor.

As shown in **Figure 3**, the related indicators can be further systematically classified into 4 categories: (left 212 jump + right 212 jump), (left single-leg jump + right single-leg jump), (double jump + triple jump), and (standing long jump + hands-on-hips long jump). Additionally, since both the 212 jump and the single-leg jump primarily assess the ability to perform single-leg jumps, these two indicators were combined for evaluation. Specifically, the evaluation was carried out using the formula (left 212 jump−left single−leg jump)left single−leg jump, with a similar calculation applied to the right side. To eliminate potential sign differences, the absolute value approach is used, providing a more reliable reflection of the single-leg jump capability. As a result, (left 212 jump + right 212 jump) and (left single-leg jump + right single-leg jump) were consolidated into a single factor belonged to impact control ability.

In evaluating upper extremity ability, we emphasize the critical role of bilateral balance control. Our analysis reveals that asymmetries in left and right hand reach length may significantly influence upper limb balance regulation. This finding underscores the importance of considering bilateral coordination in comprehensive physical assessments.

Building upon this assessment framework, we developed a systematic approach for reclassifying and integrating additional performance indicators. By applying standardized criteria, we generated novel composite metrics that more accurately describe functional capabilities across different body regions. These refined metrics, which follow established rules for variable derivation, are presented in **Table 2**. This reorganization enables a more nuanced understanding of physical performance while maintaining methodological rigor in our assessment protocol.

**Table 2:** Evaluation rules for new variables.

Variable	Rule
Upper limb balance control	Lleft hand lengthLright hand length
Lower limb balance control	Lleft leg lengthLright leg length
Core-mediated transfer control	Dstanding long jumpDhands−on−hips long jump
Explosive control	Dtriple jumpDdouble jump
Impact control	|Dleft 212 jump−Dleft single leg jump|Dleft single leg jump
	|Dright 212 jump−Dright single leg jump|Dright single leg jump

The impact control evaluation metric incorporates bilateral components, necessitating data synthesis to create a unified measure. To achieve this, we applied PCA to integrate the left and right components into a single composite variable. As shown in **Figure 4A**, the PCA yielded a principal component explaining 79.40% of the total variance. The effectiveness of this dimensional reduction is further supported by the PCA transformation results presented in **Figure 4B**, which demonstrate that the synthesized variable retains the essential information from the original bilateral measurements. This high variance retention rate confirms the robustness of our PCA approach in creating a representative composite metric for impact control assessment.

**Figure 4:**
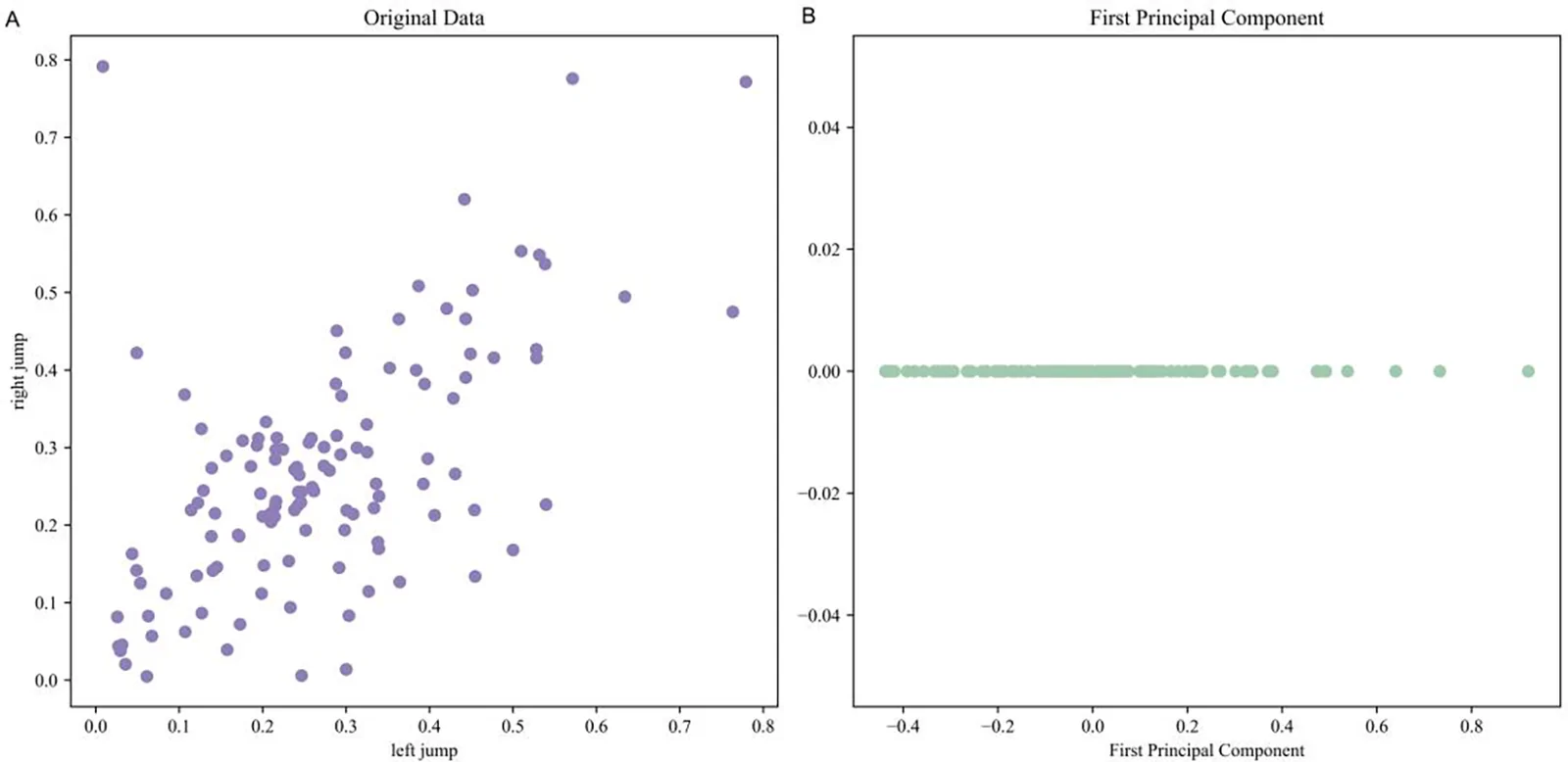
PCA transformation result. **(A)** original data’s scatter of left jump and right jump, **(B)** the first principal component.

#### 3.2.2 Comprehensive score calculation and evaluation

##### 3.2.2.1 EWM calculates the score

Based on the rule reconstruction and PCA dimensionality reduction, we obtained 5 novel variables. The next step was to compute the overall scores for these 5 variables, aiming to provide a comprehensive and rational evaluation of the FC indicators. To achieve this, we employed the EWM for the integrated assessment.

The weight distribution for each indicator is as follows: upper limb balance control (0.1998), lower limb balance control (0.2006), core-mediated transfer control (0.2027), explosive control (0.2025), and impact control (0.1945). Notably, core-mediated transfer control plays the most significant role in determining FC, while upper limb balance control and impact control contribute comparatively less. Ultimately, we derived the initial composite score.

##### 3.2.2.2 Results output and evaluation

The final calculated composite score intervals are shown in **Table 3**. For a more intuitive representation of the results, the initial composite scores were adjusted and converted into a percentage scale.

**Table 3:** Composite score intervals.

Evaluation indicator	Poor	Pass	Good	Excellent	Outstanding
Count	3	35	70	13	2

The density distribution of the composite scores is shown in **Figure 5A**. As depicted, the majority of participants’ FC fall within the pass and good ranges, with a slight rightward skew in the distribution.

**Figure 5:**
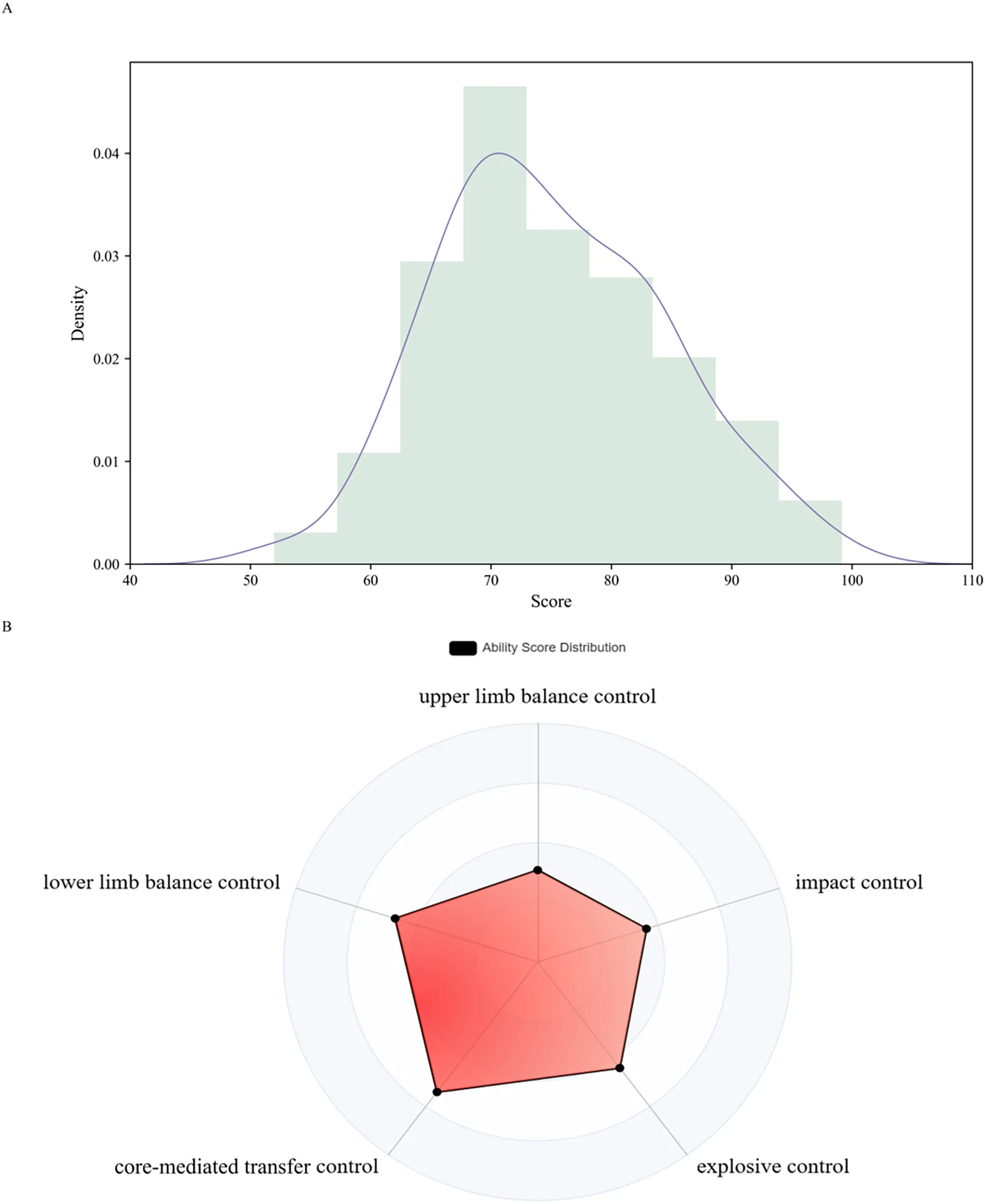
FC assessment results. **(A)** composite score’s density profile, **(B)** radar graph of median composite scores for all subjects.

The radar chart of the median scores for each component is shown in **Figure 5B**. Based on the weightings assigned to each component, the control ability scores for each participant were calculated, and the median scores for each component were plotted on a radar chart to provide a comprehensive evaluation and analysis of the participants’ physical fitness, followed by relevant recommendations. The results indicate that the overall FC of the university students is relatively balanced, with stronger abilities in core-mediated transfer control and lower limb balance control. However, upper limb balance control and impact control abilities are comparatively weaker.

## 4 Discussion

In this study, we developed a comprehensive and scientifically grounded evaluation method for college students’ FC based on select indicators of the FCS, incorporating 4 mainstream unsupervised learning algorithms to optimize the theoretical framework of FCS.

### 4.1 Relationships between the results of FA and hierarchical clustering

The FA model constructed in this study indicates that college students’ FC can be comprehensively assessed across 3 dimensions: jumping ability, upper body strength, and lower body strength. Hierarchical cluster analysis of the variables within the jumping ability factor revealed that this dimension can be further refined for more detailed evaluation. Previous studies have demonstrated the efficacy of FA in evaluating motor skills and physical capabilities (Jiang et al., 2019; Ng and Button, 2023), and this study further validates the use of related indicators to assess FC across these 3 dimensions. However, in many studies, the FA results are typically treated as the final evaluation (Lafave et al., 2016; Winters et al., 2020). When factors contain numerous variables, this can lead to imprecise results. To address this issue, this study built upon the FA analysis by conducting a deeper exploration of the jumping ability factor. Hierarchical clustering was employed to compute the distances between variables, thereby identifying more granular dimensions for FC assessment. The results from the hierarchical clustering tree provide valuable insights for further analysis.

### 4.2 Algorithms application

From the perspective of algorithm application, we utilized a stepwise analysis involving 4 unsupervised learning algorithms. Unlike previous studies, which often compared the effectiveness of multiple algorithms (Valle et al., 2022; Bruce et al., 2024; Schumacher et al., 2024) or relied on 1 or 2 machine learning algorithms for single-task analysis (Castellanos et al., 2021; Zhan et al., 2023), our study adopted 4 algorithms in succession—a rare approach in the field of sports science. The successful implementation of this multi-algorithm strategy demonstrates its potential for broader application in physical fitness assessment and related domains.

### 4.3 Comparison with FCS

The primary differences between this study and the original FCS testing are as follows: (1) Movement control is subdivided into upper limb balance control and lower limb balance control; (2) the postural control parameter is excluded because it is a multi-factorial construct (sensory, cognitive, experiential) that cannot be validly captured by simple reach or jump metrics (Chapman et al., 2008; Prosperini et al., 2016; Lim et al., 2019); (3) a core-related dimension is assessed using the ratio of standing long jump to hands-on-hips long jump, interpreted as a proxy for the core-mediated contribution of arm swing to jump performance rather than a pure isolated core-mediated transfer control measure (Tse et al., 2005; Kibler et al., 2006; Hibbs et al., 2008); (4) explosive control is evaluated using the triple jump and double jump. Drawing from the FCS method for movement control, which uses a threshold of less than 4 cm for asymmetry between sides (Li et al., 2023), this study adopts a strategy of dividing the left-side score by the right-side score for evaluating upper and lower limb balance control.

Regarding result presentation, we computed an integrated score and established 5 scoring intervals, displaying the results using radar charts for clearer visualization. In contrast to traditional motor abilities rating scales (Uwatoko et al., 2020; He et al., 2022), which rely on subjective “scoring and grading” or simplistic binary categories such as “pass or fail”, this study adopts a more data-driven approach. By leveraging machine learning algorithms, we computed a scientifically precise and accurate composite score.

In this study, the evaluation of the core-related proxy was conducted using the standing long jump and hands-on-hips long jump. The decision to incorporate a core-related dimension into the FC evaluation system was based on several key considerations: (1) Core stability is fundamental to both daily life and athletic performance, providing necessary stability and power for most physical activities; (2) core strength has long been a focal point in training. The choice of these two specific tests stems from their relationship with core-mediated energy transfer during arm swing, as highlighted by related literature (Tse et al., 2005; Kibler et al., 2006; Hibbs et al., 2008). We acknowledge, however, that the ratio is an inference rather than a direct core-mediated transfer control measurement.

In addition, this study uses the ratio of the triple jump to the double jump as a measure of explosive control. Explosive strength refers to the ability to generate maximum force in a short period, reflecting the combination of power and speed. Compared to the double jump, the triple jump requires the lower limbs to remain bent after the first two jumps and necessitates a third jump in a very short time, making it a better indicator of lower-limb explosive control. In related theoretical frameworks, the triple jump is considered an important component of lower-limb explosive strength training, with consistent practice leading to significant improvements in explosive strength. Moreover, impact control is used to assess energy storage (Li et al., 2023). This study follows the FCS theory’s approach for calculation and incorporates absolute values of the left and right leg differences to account for the rare occurrence of negative values. Additionally, PCA was applied to reduce the dimensionality of this data to a single dimension, facilitating subsequent comprehensive analysis.

### 4.4 Limitations

This study has several limitations that warrant consideration. First, for practical testing purposes, only 12 indicators derived from the FCS theoretical system were selected, and movement protocols were not further optimized. Future research could incorporate a broader set of refined and comprehensive indicators, including dedicated core endurance and core stability tests (e.g., plank, trunk stability push-up), to strengthen construct validity and further optimize the testing protocol. Second, the five evaluation rules proposed in this study may be more applicable to college students; therefore, further research is required to develop more rigorous evaluation criteria for diverse populations. Furthermore, the sample size is relatively limited for the unsupervised learning workflow used in the present study. A smaller dataset may weaken the stability of clustering outputs and the constructed evaluation framework, restricting the external generalizability of our findings. Larger sample cohorts are needed in follow-up research to verify model robustness. Finally, more advanced unsupervised learning algorithms can be adopted to further optimize the evaluation system.

## 5 Conclusion

This study systematically categorized the FC-related indicators of college students and, by integrating various unsupervised machine learning methods, innovatively established a new standard and approach for FC evaluation. Through hierarchical clustering and PCA dimensionality reduction based on FA, we re-evaluated the test indicators employed. The five newly designed evaluation rules, illustrated through the density distribution and radar plots generated from EWM-corrected results, demonstrate that our evaluation method provides a more scientific and detailed assessment of college students’ FC levels. This methodological advancement not only provides an enhanced evaluation framework for the college students but also establishes a potential paradigm for assessing motor skills, physical abilities, and diverse population groups across related research domains.

## Data Availability

The raw data supporting the conclusions of this article will be made available by the authors, without undue reservation.
